# The status and influencing factors of lung ventilation function in employees exposed to dust in enterprises of the XPCC, China

**DOI:** 10.3389/fpubh.2024.1370765

**Published:** 2024-04-26

**Authors:** Yiman Zhao, Siqi Zhao, Jiaying Lu, Ruoyun Dong, Qianqian Wang, Guanling Song, Yunhua Hu

**Affiliations:** Department of Preventive Medicine/The Key Laboratory for Prevention and Control of Emerging Infectious Diseases and Public Health Security, The Xinjiang Production and Construction Corps, School of Medicine, Shihezi University, Shihezi, Xinjiang, China

**Keywords:** occupational health, lung ventilation function, dust exposure, influencing factor, workplace

## Abstract

**Background:**

Occupational health is closely related to harmful factors in the workplace. Dust is the primary contributing factor causing impaired lung ventilation function among employees with dust exposure, and their lung ventilation function may also be influenced by other factors. We aimed at assessing the status and influencing factors of lung ventilation function among employees exposed to dust in the enterprises of the Eighth Division located in the Xinjiang Production and Construction Corps (XPCC), China.

**Methods:**

Employees exposed to dust in enterprises of the Eighth Division located in the XPCC in 2023 were selected as the subjects of this cross-sectional study. Their lung ventilation function indicators were extracted from health examination records, and an on-site electronic questionnaire survey was conducted among them. Binary logistic regression analyses were conducted to evaluate the factors influencing lung ventilation function.

**Results:**

According to the fixed value criteria, the abnormal rates of forced expiratory volume in 1 s (FEV_1_), forced vital capacity (FVC), and FEV_1_/FVC were 31.6, 1.4, and 0.4%, respectively. The lower limit of normal (LLN) criteria could overestimate the rate of abnormal lung ventilation function. Several factors were related to impaired lung ventilation function, including gender, age, education level, marital status, body mass index (BMI), smoking status, physical activity, the type of dust, industry, enterprise scale, occupation, length of service, working shift, monthly income, and respiratory protection.

**Conclusions:**

A relatively low abnormal rate of lung ventilation function was observed among employees exposed to dust in enterprises of the Eighth Division, XPCC, and their lung ventilation function was associated with various factors. Effective measures should be taken urgently to reduce the effects of adverse factors on lung ventilation function, thereby further protecting the health of the occupational population.

## 1 Introduction

Occupational harmful factors are known to be diverse and widespread (1), and employees may be exposed to these factors during work (2). Compared to the general population, occupational populations are exposed to higher levels of adverse factors (3). This suggests that the health status of occupational populations may be more vulnerable to occupational exposures. Mason et al. pointed out that occupational asthma could be induced by the inciting agents in the workplace, including sensitizers and irritants (4). In addition, occupational exposure to diisocyanate could trigger asthma, with males being at higher risk (5). Campisi et al. have pointed out that occupational exposure is closely associated with the development of chronic obstructive pulmonary disease (6). Therefore, the health status of the occupational populations deserves focused attention.

Dust is one of the most common harmful factors in the working environment. According to the World Health Organization (WHO), ~125 million people worldwide are exposed to asbestos in the workplace (7). In Finland, the number of people exposed to asbestos in 2019 almost tripled compared to 2010 (8). One study showed that the number of deaths due to occupational asbestos in China was 24,264 in 2017, which represented a 318.7% increase compared to 1990 (9). It can be seen that dust exposure involves a large and increasing number of employees and may pose significant risks to their health, which has raised concerns among the public about the health of employees exposed to dust in enterprises.

It is widely recognized that the respiratory system is the first area to be affected after dust inhalation, causing respiratory diseases (10). Some scholars have pointed out that particulate matter can stimulate the respiratory tract or cause allergic reactions, thereby promoting the occurrence of asthma (11). A 20-year follow-up study has shown that long-term occupational exposure to dust can accelerate the decline of forced expiratory volume in 1 s (FEV_1_) and the ratio of FEV_1_ to forced vital capacity (FVC), with the latter expressed as FEV_1_/FVC (12). In addition, the advancement of science and technology has spurred the emergence of new industries, along with the generation of novel dust types, which could also damage the lung health of workers. For instance, Guarnieri et al. discovered that the production of new high-silica-content artificial stone, known as quartz conglomerates, could lead to the onset of silicosis (13). Similarly, workers exposed to artificial quartz conglomerates in the finishing sector exhibited a higher incidence of silicosis (14). Therefore, it is urgent to take effective measures to achieve early detection, diagnosis, and treatment of lung ventilation function damage in employees exposed to dust, thereby protecting the health of the occupational population.

Lung function parameters are considered important and reliable indicators for assessing normal and abnormal states of the airways (15). FVC, FEV_1_, and FEV_1_/FVC are the most common indicators in the studies on the influencing factors of lung function, which can better reflect lung ventilation function. FVC can reflect the expiratory resistance of large airways (16). FEV_1_ is the main indicator of impaired lung function (17). FEV_1_/FVC is commonly used to identify obstructive or restrictive damage (18). Regular lung ventilation function tests are beneficial for early detection of lung ventilation function abnormalities and prevention of lung-related diseases in employees exposed to dust, especially those in the industrial domain.

In recent years, with the continuous advancement of China's industrialization process, many small mines, factories, and heavy metal industries have emerged, resulting in a large number of employees being exposed to dust (19). As a hub of national energy and other resources, Xinjiang is highly dependent on resource development, and heavy industry occupies a dominant position in the industrial structure (20), which indicates that the lung ventilation function of the population in this area, especially employees exposed to dust, may be affected. In 2019, the Health Xinjiang Action for 2019–2030 was developed (21), and the Occupational Health Protection Action was one of the major thematic actions that aimed to protect the health status of occupational groups in Xinjiang.

Since the reform and opening up, the Xinjiang Production and Construction Corps (XPCC) industry has become an important force in Xinjiang's industry. Available data show that the Eighth Division ranks first in the XPCC in terms of the number of industrial enterprises above the scale and the number of employees (22). This suggests that the number of employees exposed to dust may be higher in the Eighth Division compared to other divisions and regiments in the XPCC, and their lung ventilation function is obviously worthy of attention. Therefore, this study aims to describe the lung ventilation function status of the employees exposed to dust in enterprises located in the Eighth Division of the XPCC in 2023 and to explore its influencing factors, thus providing a theoretical basis for the protection of lung ventilation function among employees exposed to dust in enterprises located in this region.

## 2 Methods

### 2.1 Study participants and data collection

Employees exposed to dust who had participated in at least one lung ventilation function test in enterprises of the Eighth Division located in the XPCC in 2023 were selected as the subjects of this study. The subjects were screened prior to this study. Firstly, a total of 43,765 health examination records of employees exposed to dust were collected from the Occupational Disease and Occupational Health Information Monitoring System between January 1, 2023 and December 31, 2023. Secondly, we excluded: (1) pre-employment examiners; (2) length of service <1 year; (3) no lung ventilation function outcome; (4) no occupation; and (5) duplicate records. Notably, if an individual underwent multiple health examinations during this period, the first record was retained. The employees were exposed to different types of dust, including coal dust, silica dust, polyvinyl chloride (PVC) dust, and other dusts. Of these, other dusts primarily consisting of unidentified types of dust and a small amount of organic and inorganic dust. In addition, the concentrations of dust to which all individuals were exposed did not exceed the occupational exposure limits.

An on-site electronic questionnaire survey was carried out among them in 2023, which retrospectively collected information on their general demographic, behavioral, and occupational characteristics, as well as the history of respiratory and cardiovascular diseases. Notably, individuals with any of the following conditions were not eligible to participate in the survey: (1) having a history of mental illness; (2) currently receiving psychological treatment or taking anti-psychotic medication; and (3) refusing to participate in the survey. In the end, a total of 9,641 questionnaires were obtained. After that, duplicate records were removed and the final record of questionnaire was selected. Notably, all individuals included in this study took more than 3 min to complete the questionnaire without submitting it prematurely, indicating that the data from the questionnaire was more reliable.

**Figure 1:**
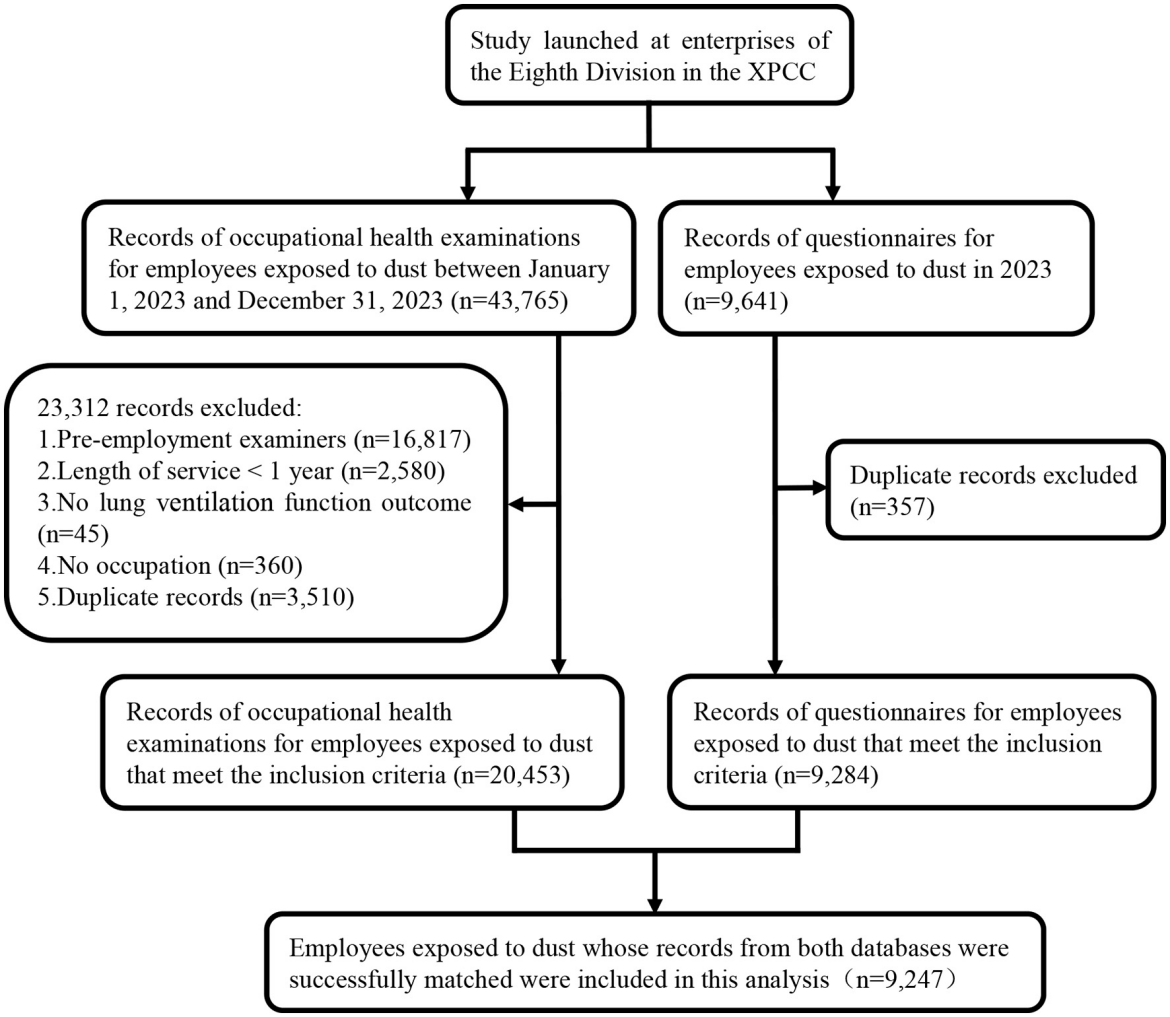
Flow chart of the enrolled subjects.

Subsequently, the records in both databases were combined based on their ID numbers. Ultimately, 9,247 participants exposed to dust with no missing data were included in this analysis. The specific screening process is shown in Figure 1. This study protocol was approved by the Science and Technology Ethical Committee of the First Affiliated Hospital, School of Medicine, Shihezi University (Ethical Review Number: KJX2022-104-01). Informed consent was obtained from all participants before enrolling in the survey.

### 2.2 Quality control

Standardized questionnaires were used as references when developing the questionnaire (23–27). The questionnaire was evaluated through a pre-survey, and expert advice was sought to improve the original questionnaire. Harmonized training was attended by the investigators prior to the survey. Finally, data cleaning was employed to further ensure the quality of the data from two databases.

### 2.3 Lung ventilation function test

According to the standardization of spirometry updated by the ATS/ERS Task Force in 2019 (28), lung ventilation function tests were conducted on participants by well-trained health technicians using body plethysmography (Elite DX; MedGraphics Corp., Saint Paul, MN, USA). The health technicians took into account all the contraindications in advance. After calibrating the spirometer, the participants were instructed to wear a nose clip correctly, inhale as deeply as possible until no more air could be inhaled, then insert the mouthpiece within 2 s, seal their lips around the mouthpiece, and begin maximal exhalation. All measurements were collected at least three times in duplicate, and the maximum value of the measurement was recorded. Lung ventilation function indices included FVC, FEV_1_, and FEV_1_/FVC, and their results were expressed as a percentage of the predicted values (namely FVC% pred, FEV_1_% pred, and FEV_1_/FVC% pred) by dividing the lung ventilation function measured values by the predicted values. It is worth noting that the predicted values could be calculated on the basis of factors such as gender, age, and height.

There were two distinctive approaches utilized to evaluate lung ventilation function: the fixed value criteria and the lower limit of normal (LLN) criteria. According to the fixed value criteria (29), both FEV_1_% pred and FVC% pred were defined as normal when they measured ≥80%, while an FEV_1_/FVC% pred ≥70% was considered normal.

Due to variations in lung ventilation function based on factors such as gender, the ATS/ERS Task Force recommended using the measured value and LLN to assess for abnormalities in indicators of lung ventilation function (30). Therefore, the predicted values and LLN for FEV_1_, FVC, and FEV_1_/FVC were calculated using the Global Lung Function Initiative reference equations (31). Subsequently, the measured values of lung ventilation function indices were calculated based on the percentages of the predicted values and the predicted values. In this study, when the measured values of FEV_1_, FVC, and FEV_1_/FVC were lower than their corresponding LLN, they were considered abnormal; otherwise, they were considered normal.

### 2.4 Exposure variables

Among these exposure variables, gender, height, weight, industry, enterprise scale, occupation, length of service, and the type of dust were derived from the records of health examinations. The ages of the participants were calculated with the date of examination and birth. Other relevant factors, such as educational level, were all self-reported in the questionnaire.

General demographic factors included gender (male, female), age (≤50, >50 years), education level (junior college or above, high school or technical secondary school, junior high school or below), marital status (married, unmarried, divorced or widowed), and body mass index (BMI). The BMI was calculated with height and weight. The participants were categorized as underweight (BMI < 18.5 kg/m^2^), normal weight (18.5 kg/m^2^ ≤ BMI < 24.0 kg/m^2^), overweight (24.0 kg/m^2^ ≤ BMI < 28.0 kg/m^2^), and obese (BMI ≥ 28.0 kg/m^2^) based on BMI (32).

Behavioral factors involved smoking status, alcohol consumption, and physical activity. According to the criteria of the WHO (33), people were categorized as current smokers, ex-smokers, and never-smokers. Based on the smoking index (SI), current smokers were further categorized into light smokers (SI ≤ 400) and heavy smokers (SI > 400) (34). The smoking index was equal to the average number of cigarettes smoked per day multiplied by the number of years smoked (35). Alcohol consumption was classified as current drinkers and non-drinkers (past/never drinkers) (27). The physical activity was investigated with the International Physical Activity Questionnaire-Short Version (IPAQ-SV) and divided into three levels, including high, moderate, and low (24).

Occupational factors included the type of dust, industry (mining, manufacturing, and other industries), enterprise scale (large or medium enterprise, small or micro enterprise), occupation (mixed, mental, and physical laborer), length of service ( ≤5, 5–10, >10 years), working shift (day shifts, night or rotating shifts), monthly income (<5,000, 5,000–9,999, ≥10,000 RMB), and the presence of respiratory protection (yes, no). It was important to note that the types of dust included coal dust, silica dust, PVC dust, and other dusts, with the latter primarily consisting of unidentified types of dust and a small amount of organic and inorganic dust. Other industries include the primary and tertiary industries, as well as the production and supply of electricity, heat, gas, and water in the secondary industry.

In addition, the history of respiratory diseases (yes, no) and cardiovascular diseases (yes, no) was also self-reported. Among them, respiratory diseases involved upper respiratory tract infections, chronic bronchitis, chronic sinusitis, bronchial asthma, emphysema, tuberculosis, and other respiratory diseases. Cardiovascular diseases included hypertension, coronary heart disease, myocardial infarction, myocardial insufficiency, and other cardiovascular diseases.

### 2.5 Statistical analysis

Data cleaning and analysis were conducted with SPSS 26.0 statistical software in this research. Count, rate, and percentage were used to describe categorical variables. Continuous variables were described as mean ± standard deviation (SD). Comparisons between the rates of abnormal lung ventilation function in the different groups of participants were tested by the χ^2^-test. Multicollinearity was detected, and there was no multicollinearity among the exposure variables. Subsequently, factors that were statistically significant or clinically considered to be closely related to lung ventilation function were considered independent variables in the logistic regression analyses. Three regression models were developed to estimate the relationship between lung ventilation function and exposure variables. Model 1 was adjusted for gender and age. Model 2 was additionally adjusted for education level, marital status, BMI, smoking status, alcohol consumption, and physical activity on the basis of Model 1. Model 3 built on Model 2 and additionally adjusted working shift, respiratory protection, respiratory disease history, and cardiovascular disease history. Compared to Models 1 and 2, Model 3 more accurately captures the true impact of the independent variables on the dependent variable. The odds ratios (ORs) and 95% confidence interval (95% CI) were calculated to assess the risk of abnormal lung ventilation function in different groups of employees exposed to dust. A *P*-value of <0.05 indicates that the difference is statistically significant.

## 3 Results

### 3.1 Basic characteristics of study participants

As shown in Table 1, a total of 9,247 study participants were ultimately included in this analysis, of whom 78.1% were male and 21.9% were female. People aged ≤ 50 (90.1%), married individuals (67.1%), those with an abnormal BMI (BMI < 18.5 kg/m^2^ or BMI ≥ 24.0 kg/m^2^; 58.1%), never-smokers (55.4%), non-drinkers (73.7%), and those engaged in low levels of physical activity (42.8%) were more common among the subjects. Among all participants, 4,449 individuals had a junior college education or above (48.1%).

**Table 1:** Basic characteristics of the study population (*N* = 9,247).

**Variables**	**Number (*n*)**	**Frequency (%)**
**Gender**		
Female	2,021	21.9
Male	7,226	78.1
**Age (years)**		
≤ 50	8,331	90.1
>50	916	9.9
**Education level**		
Junior college or above	4,449	48.1
High school or technical secondary school	2,749	29.7
Junior high school or below	2,049	22.2
**Marital status**		
Married	6,208	67.1
Unmarried	2,621	28.4
Divorced or widowed	418	4.5
**BMI**		
Underweight	257	2.8
Normal weight	3,876	41.9
Overweight	3,509	37.9
Obese	1,605	17.4
**Smoking status**		
Never-smokers	5,123	55.4
Ex-smokers	626	6.8
Light smokers	3,402	36.8
Heavy smokers	96	1.0
**Alcohol consumption**		
Non-drinkers	6,814	73.7
Current drinkers	2,433	26.3
**Physical activity**		
Low	3,958	42.8
Moderate	2,017	21.8
High	3,272	35.4
**Type of dust**		
Coal dust	2,139	23.1
Silica dust	2,446	26.5
PVC dust	351	3.8
Other dusts	4,311	46.6
**Industry**		
Mining	103	1.1
Manufacturing	8,315	89.9
Other industries	829	9.0
**Enterprise scale**		
Large or medium	7,062	76.4
Small or micro	2,185	23.6
**Occupation**		
Mixed laborer	3,326	36.0
Mental laborer	225	2.4
Physical laborer	5,696	61.6
**Length of service (years)**		
≤ 5	3,944	42.6
5–10	2,394	25.9
>10	2,909	31.5
**Working shift**		
Day shifts	3,186	34.5
Night or rotating shifts	6,061	65.5
**Monthly income (RMB)**		
< 5,000	3,567	38.6
5,000–9,999	5,465	59.1
≥10,000	215	2.3
**Respiratory protection**		
Yes	7,483	80.9
No	1,764	19.1
**Respiratory diseases history**		
Yes	2,539	27.5
No	6,708	72.5
**Cardiovascular diseases history**		
Yes	2,328	25.2
No	6,919	74.8

BMI, body mass index, PVC dust, polyvinyl chloride dust.

Regarding the types of dust, individuals exposed to coal dust and silica dust accounted for 23.1 and 26.5% of the total, respectively. The vast majority of employees were employed in manufacturing (89.9%). The number of employees in large or medium enterprises (76.4%) was higher than that in small or micro enterprises (23.6%). Physical laborers (61.6%), employees with ≤5 years of service (42.6%), night or rotating shifts individuals (65.5%), those earning 5,000–9,999 RMB (59.1%), and those with respiratory protection (80.9%) were the most prevalent within the subjects. Approximately 27.5% of employees had a history of respiratory diseases, and 25.2% of employees had a history of cardiovascular diseases.

### 3.2 Univariate analysis

The mean (± SD) of FEV_1_ was 85.66% (± 11.32). The mean (± SD) of FVC was 88.14% (± 10.57). For the FEV_1_/FVC, the mean (± SD) was 97.52% (± 10.94). According to the fixed value criteria, the prevalence of abnormal FEV_1_, FVC, and FEV_1_/FVC was 31.6, 1.4, and 0.4%, respectively, which was lower than the rates of abnormality in FEV_1_ (50.7%), FVC (55.4%), and FEV_1_/FVC (23.2%) based on the LLN criteria (Table 2).

**Table 2:** The prevalence of abnormal lung ventilation function based on two criteria.

**Variables**	**Mean ±SD**	**LLN criteria (%)**	**Fixed value criteria (%)**
FEV_1_ (% pred)	85.66 ± 11.32	50.7	31.6
FVC (% pred)	88.14 ± 10.57	55.4	1.4
FEV_1_/FVC (% pred)	97.52 ± 10.94	23.2	0.4

FVC, forced vital capacity; FEV_1_, forced expiratory volume in 1 s; SD, standard deviation; LLN, lower limit of normal.

Results of the χ^2^-tests are summarized in Table 3. The occurrence rates of abnormal FEV_1_/FVC (*P* = 0.006) varied among employees of different ages, and individuals aged > 50 had a higher prevalence of abnormal FEV_1_/FVC compared to those aged ≤ 50. The statistical analysis revealed significant variations in the prevalence of abnormal FEV_1_ (*P* = 0.001), FVC (*P* < 0.001), and FEV_1_/FVC (*P* < 0.001) across employees of different educational levels. As education level decreased, there was an upward trend in the abnormal rates of FEV_1_ and FVC, while a downward trend was observed in FEV_1_/FVC.

**Table 3:** The prevalence of abnormal FEV_1_, FVC, and FEV_1_/FVC among employees with different features.

**Variables**	**FEV_1_ (*n*, %)**	**χ^2^**	***P***	**FVC (*n*, %)**	**χ^2^**	***P***	**FEV_1_/FVC (*n*, %)**	**χ^2^**	***P***
	**Abnormal**			**Abnormal**			**Abnormal**		
**Gender**									
Female	776 (38.4)	157.387	**< 0.001**	851 (42.1)	184.055	**< 0.001**	499 (24.7)	3.051	0.081
Male	3,915 (54.2)			4,269 (59.1)			1,650 (22.8)		
**Age (years)**									
≤ 50	4,239 (50.9)	0.780	0.377	4,611 (55.3)	0.016	0.899	1,903 (22.8)	7.452	**0.006**
>50	452 (49.3)			509 (55.6)			246 (26.9)		
**Education level**									
Junior college or above	2,170 (48.8)	14.393	**0.001**	2,289 (51.4)	65.720	**< 0.001**	1,219 (27.4)	84.911	**< 0.001**
High school or technical secondary school	1,425 (51.8)			1,562 (56.8)			552 (20.1)		
Junior high school or below	1,096 (53.5)			1,269 (61.9)			378 (18.4)		
**Marital status**									
Married	2,997 (48.3)	54.829	**< 0.001**	3,329 (53.6)	23.487	**< 0.001**	1,451 (23.4)	1.745	0.418
Unmarried	1,490 (56.8)			1,549 (59.1)			612 (23.3)		
Divorced or widowed	204 (48.8)			242 (57.9)			86 (20.6)		
**BMI**									
Underweight	143 (55.6)	26.530	**< 0.001**	157 (61.1)	47.705	**< 0.001**	53 (20.6)	2.156	0.541
Normal weight	1,868 (48.2)			1,995 (51.5)			886 (22.9)		
Overweight	1,791 (51.0)			1,997 (56.9)			822 (23.4)		
Obese	889 (55.4)			971 (60.5)			388 (24.2)		
**Smoking status**									
Never-smokers	2,433 (47.5)	49.418	**< 0.001**	2,653 (51.8)	60.373	**< 0.001**	1,230 (24.0)	14.685	**0.002**
Ex-smokers	330 (52.7)			365 (58.3)			154 (24.6)		
Light smokers	1,875 (55.1)			2,044 (60.1)			732 (21.5)		
Heavy smokers	53 (55.2)			58 (60.4)			33 (34.4)		
**Alcohol consumption**									
Non-drinkers	3,386 (49.7)	11.167	**0.001**	3,701 (54.3)	11.657	**0.001**	1,574 (23.1)	0.286	0.593
Current drinkers	1,305 (53.6)			1,419 (58.3)			575 (23.6)		
**Physical activity**									
Low	1,999 (50.5)	13.944	**0.001**	2,220 (56.1)	13.101	**0.001**	923 (23.3)	11.462	**0.003**
Moderate	961 (47.6)			1,046 (51.9)			518 (25.7)		
High	1,731 (52.9)			1,854 (56.7)			708 (21.6)		
**Type of dust**									
Coal dust	1,115 (52.1)	10.100	**0.018**	1,167 (54.6)	32.804	**< 0.001**	611 (28.6)	160.399	**< 0.001**
Silica dust	1,260 (51.5)			1,457 (59.6)			396 (16.2)		
PVC dust	196 (55.8)			214 (61.0)			30 (8.5)		
Other dusts	2,120 (49.2)			2,282 (52.9)			1,112 (25.8)		
**Industry**									
Mining	66 (64.1)	12.626	**0.002**	84 (81.6)	79.998	**< 0.001**	15 (14.6)	366.044	**< 0.001**
Manufacturing	4,237 (51.0)			4,677 (56.2)			1,720 (20.7)		
Other industries	388 (46.8)			359 (43.3)			414 (49.9)		
**Enterprise scale**									
Large or medium	3,466 (49.1)	32.569	**< 0.001**	3,693 (52.3)	114.383	**< 0.001**	1,600 (22.7)	5.704	**0.017**
Small or micro	1,225 (56.1)			1,427 (65.3)			549 (25.1)		
**Occupation**									
Mixed laborer	1,556 (46.8)	33.598	**< 0.001**	1,641 (49.3)	78.032	**< 0.001**	1,066 (32.1)	231.736	**< 0.001**
Mental laborer	111 (49.3)			123 (54.7)			56 (24.9)		
Physical laborer	3,024 (53.1)			3,356 (58.9)			1,027 (18.0)		
**Length of service (years)**									
≤ 5	2,182 (55.3)	81.319	**< 0.001**	2,355 (59.7)	89.583	**< 0.001**	804 (20.4)	68.484	**< 0.001**
5–10	1,220 (51.0)			1,358 (56.7)			514 (21.5)		
>10	1,289 (44.3)			1,407 (48.4)			831 (28.6)		
**Working shift**									
Day shifts	1,549 (48.6)	8.666	**0.003**	1,749 (54.9)	0.440	0.507	820 (25.7)	16.998	**< 0.001**
Night or rotating shifts	3,142 (51.8)			3,371 (55.6)			1,329 (21.9)		
**Monthly income (RMB)**									
< 5,000	1,663 (46.6)	45.747	**< 0.001**	1,768 (49.6)	88.728	**< 0.001**	1,056 (29.6)	136.716	**< 0.001**
5,000–9,999	2,895 (53.0)			3,203 (58.6)			1,065 (19.5)		
≥10,000	133 (61.9)			149 (69.3)			28 (13.0)		
**Respiratory protection**									
Yes	3,792 (50.7)	0.048	0.827	4,098 (54.8)	5.814	**0.016**	1,728 (23.1)	0.479	0.489
No	899 (51.0)			1,022 (57.9)			421 (23.9)		
**Respiratory diseases history**									
Yes	1,346 (53.0)	7.299	**0.007**	1,421 (56.0)	0.506	0.477	611 (24.1)	1.334	0.248
No	3,345 (49.9)			3,699 (55.1)			1,538 (22.9)		
**Cardiovascular diseases history**									
Yes	1,236 (53.1)	6.949	**0.008**	1,348 (57.9)	8.087	**0.004**	553 (23.8)	0.461	0.497
No	3,455 (49.9)			3,772 (54.5)			1,596 (23.1)		

BMI, body mass index; FVC, forced vital capacity; FEV_1_, forced expiratory volume in 1 s; PVC dust, polyvinyl chloride dust.

Bold indicates that the *P*-value is < 0.05, indicating statistical significance.

The abnormal rates of FEV_1_ and FVC were associated with gender, marital status, and BMI (all *P* < 0.001). The abnormality rates of FEV_1_ and FVC were higher in males than in females. Compared to married individuals, the rates of abnormal FEV_1_ and FVC increased among unmarried individuals and divorced or widowed individuals. Furthermore, the prevalence of abnormal FEV_1_ and FVC was higher among employees in the underweight, overweight, and obese groups compared to the normal weight group, with the underweight group exhibiting the highest rates of abnormal FEV_1_ and FVC.

There was a significant association between the rates of abnormal lung ventilation function and smoking status, and both ex-smokers and heavy smokers exhibited higher rates of abnormal FEV_1_, FVC, and FEV_1_/FVC compared to never-smokers. The abnormality rates of FEV_1_ and FVC were higher in current drinkers than in non-drinkers (all *P* = 0.001). The abnormality rates of FEV_1_ (*P* = 0.001), FVC (*P* = 0.001), and FEV_1_/FVC (*P* = 0.003) varied among employees of different levels of physical activity, and individuals engaging in moderate physical activity exhibited a significantly lower rate of abnormal FEV_1_ and FVC than those engaging in low physical activity.

The prevalence of abnormal FEV_1_, FVC, and FEV_1_/FVC was statistically associated with the type of dust, industry, enterprise scale, occupation, length of service, and monthly income (all *P* < 0.05). Compared to individuals exposed to coal dust, those exposed to silica dust and PVC dust exhibited increased rates of abnormal FVC, while those exposed to other dusts demonstrated a decreased rate of abnormal FEV_1_, FVC, and FEV_1_/FVC. In addition, the abnormality rates of FEV_1_ and FVC among employees in mining were higher than those in manufacturing and other industries. With respect to enterprise scale, individuals employed in small or micro enterprises were more likely to have higher rates of abnormal FEV_1_, FVC, and FEV_1_/FVC than those in large or medium enterprises. Mental laborers and physical laborers had higher rates of abnormal FEV_1_ and FVC than mixed laborers. Additionally, the prevalence of abnormal FEV_1_ and FVC increased with higher monthly income and a shorter length of service.

Regarding the working shift, the rate of FEV_1_ abnormalities was significantly higher in employees who worked night or rotating shifts than in those who worked day shifts (*P* = 0.003), while the opposite was true for FEV_1_/FVC (*P* < 0.001). Notably, the absence of respiratory protection could significantly increase the rate of abnormal FVC (*P* = 0.016). In addition, the abnormal rate of FEV_1_ was significantly higher in employees who had a history of respiratory diseases than those without a history of respiratory diseases (*P* = 0.007). Employees with a history of cardiovascular diseases had significantly higher rates of FEV_1_ (*P* = 0.008) and FVC (*P* = 0.004) abnormalities than those without a history of cardiovascular diseases.

### 3.3 Multivariate analysis

The results of the logistic regression analyses for FEV_1_, FVC, and FEV_1_/FVC are presented in Tables 4–6, respectively. Multiple factors were found to have a significant impact on all three lung ventilation function indicators, including gender, age, education level, physical activity, occupation, working shift, and monthly income. Regarding gender, males had higher abnormal risks of FEV_1_ and FVC than females, with ORs of 1.788 (1.586–2.016) and 1.895 (1.681–2.137) in Model 3, respectively, while they had a lower risk of abnormal FEV_1_/FVC with an OR of 0.870 (0.758–1.000) in Model 3. It was confusing that, compared to employees aged ≤ 50, those aged > 50 [0.861 (0.743–0.997)] had a decreased risk of abnormal FEV_1_ in Model 2. Similarly, for FVC, the corresponding OR was 0.816 (0.703–0.948) in Model 3. Conversely, for FEV_1_/FVC, the corresponding OR was 1.445 (1.219–1.713) in Model 3, indicating a higher risk of FEV_1_/FVC abnormalities.

**Table 4:** The risk of abnormal FEV_1_ among employees with different features.

**Variables**	**Model 1**			**Model 2**			**Model 3**		
	**OR**	**95% CI**	***P***	**OR**	**95% CI**	***P***	**OR**	**95% CI**	***P***
**Gender**									
Female	1.000			1.000			1.000		
Male	1.918	1.733–2.122	**< 0.001**	1.772	1.573–1.997	**< 0.001**	1.788	1.586–2.016	**< 0.001**
**Age (years)**									
≤ 50	1.000			1.000			1.000		
>50	0.860	0.750–0.988	**0.033**	0.861	0.743–0.997	**0.046**	0.863	0.744–1.000	0.050
**Education level**									
Junior college or above	1.000			1.000			1.000		
High school or technical secondary school	1.114	1.011–1.227	**0.029**	1.173	1.062–1.296	**0.002**	1.156	1.046–1.277	**0.005**
Junior high school or below	1.264	1.134–1.410	**< 0.001**	1.361	1.216–1.523	**< 0.001**	1.319	1.177–1.479	**< 0.001**
**Marital status**									
Married	1.000			1.000			1.000		
Unmarried	1.317	1.198–1.448	**< 0.001**	1.389	1.258–1.533	**< 0.001**	1.370	1.240–1.513	**< 0.001**
Divorced or widowed	0.998	0.817–1.218	0.981	0.961	0.786–1.175	0.699	0.963	0.788–1.179	0.718
**BMI**									
Normal weight	1.000			1.000			1.000		
Underweight	1.535	1.186–1.988	**0.001**	1.485	1.144–1.926	**0.003**	1.467	1.131–1.904	**0.004**
Overweight	1.034	0.942–1.136	0.478	1.066	0.970–1.172	0.182	1.066	0.970–1.172	0.182
Obese	1.232	1.094–1.386	**0.001**	1.243	1.103–1.400	**< 0.001**	1.241	1.101–1.398	**< 0.001**
**Smoking status**									
Never-smokers	1.000			1.000			1.000		
Ex-smokers	0.998	0.840–1.186	0.984	1.008	0.847–1.199	0.932	1.006	0.845–1.197	0.950
Light smokers	1.074	0.974–1.183	0.153	1.070	0.966–1.185	0.198	1.077	0.972–1.193	0.156
Heavy smokers	1.162	0.767–1.758	0.479	1.191	0.784–1.809	0.412	1.194	0.785–1.814	0.407
**Alcohol consumption**									
Non-drinkers	1.000			1.000			1.000		
Current drinkers	0.978	0.888–1.078	0.661	0.975	0.880–1.080	0.626	0.979	0.883–1.085	0.684
**Physical activity**									
Low	1.000			1.000			1.000		
Moderate	0.884	0.793–0.985	**0.026**	0.897	0.805–1.001	0.053	0.911	0.817–1.017	0.098
High	1.044	0.950–1.147	0.370	1.045	0.951–1.148	0.364	1.057	0.961–1.162	0.255
**Type of dust**									
Coal dust	1.000			1.000			1.000		
Silica dust	0.938	0.834–1.055	0.286	0.945	0.839–1.065	0.354	0.957	0.850–1.079	0.472
PVC dust	1.144	0.910–1.439	0.250	1.188	0.943–1.496	0.144	1.168	0.927–1.472	0.187
Other dusts	0.877	0.790–0.974	**0.014**	0.903	0.812–1.005	0.061	0.914	0.821–1.017	0.098
**Industry**									
Mining	1.000			1.000			1.000		
Manufacturing	0.586	0.389–0.881	**0.010**	0.648	0.429–0.978	**0.039**	0.651	0.431–0.983	**0.041**
Other industries	0.481	0.313–0.739	**0.001**	0.549	0.356–0.848	**0.007**	0.555	0.359–0.857	**0.008**
**Enterprise scale**									
Large or medium	1.000			1.000			1.000		
Small or micro	1.272	1.153–1.404	**< 0.001**	1.199	1.080–1.332	**0.001**	1.227	1.104–1.364	**< 0.001**
**Occupation**									
Mixed laborer	1.000			1.000			1.000		
Mental laborer	1.257	0.956–1.654	0.102	1.300	0.987–1.714	0.062	1.343	1.017–1.773	**0.038**
Physical laborer	1.259	1.155–1.373	**< 0.001**	1.192	1.090–1.303	**< 0.001**	1.166	1.064–1.277	**0.001**
**Length of service (years)**									
≤ 5	1.000			1.000			1.000		
5–10	0.809	0.730–0.897	**< 0.001**	0.845	0.760–0.940	**0.002**	0.846	0.760–0.941	**0.002**
>10	0.614	0.556–0.678	**< 0.001**	0.664	0.596–0.739	**< 0.001**	0.660	0.593–0.736	**< 0.001**
**Working shift**									
Day shifts	1.000			1.000			1.000		
Night or rotating shifts	1.207	1.106–1.317	**< 0.001**	1.147	1.050–1.254	**0.002**	1.149	1.051–1.257	**0.002**
**Monthly income (RMB)**									
< 5,000	1.000			1.000			1.000		
5,000–9,999	1.113	1.018–1.218	**0.019**	1.133	1.035–1.240	**0.007**	1.135	1.036–1.243	**0.006**
≥10,000	1.518	1.141–2.020	**0.004**	1.548	1.161–2.064	**0.003**	1.612	1.206–2.155	**0.001**
**Respiratory protection**									
Yes	1.000			1.000			1.000		
No	1.020	0.919–1.133	0.706	1.019	0.916–1.133	0.733	1.039	0.933–1.156	0.486

BMI, body mass index; OR, odds ratio; 95% CI, 95% confidence interval; FEV_1_, forced expiratory volume in 1 s; PVC dust, polyvinyl chloride dust.

Bold indicates that the *P*-value is < 0.05, indicating statistical significance.

**Table 5:** The risk of abnormal FVC among employees with different features.

**Variables**	**Model 1**			**Model 2**			**Model 3**		
	**OR**	**95% CI**	***P***	**OR**	**95% CI**	***P***	**OR**	**95% CI**	***P***
**Gender**									
Female	1.000			1.000			1.000		
Male	1.997	1.806–2.208	**< 0.001**	1.884	1.672–2.122	**< 0.001**	1.895	1.681–2.137	**< 0.001**
**Age (years)**									
≤ 50	1.000			1.000			1.000		
>50	0.917	0.798–1.053	0.221	0.823	0.710–0.955	**0.010**	0.816	0.703–0.948	**0.008**
**Education level**									
Junior college or above	1.000			1.000			1.000		
High school or technical secondary school	1.224	1.110–1.350	**< 0.001**	1.269	1.148–1.402	**< 0.001**	1.276	1.154–1.411	**< 0.001**
Junior high school or below	1.622	1.451–1.813	**< 0.001**	1.696	1.512–1.903	**< 0.001**	1.695	1.508–1.904	**< 0.001**
**Marital status**									
Married	1.000			1.000			1.000		
Unmarried	1.158	1.053–1.275	**0.003**	1.275	1.154–1.409	**< 0.001**	1.264	1.143–1.398	**< 0.001**
Divorced or widowed	1.159	0.947–1.420	0.153	1.092	0.890–1.341	0.398	1.093	0.890–1.341	0.398
**BMI**									
Normal weight	1.000			1.000			1.000		
Underweight	1.707	1.312–2.221	**< 0.001**	1.732	1.328–2.260	**< 0.001**	1.719	1.317–2.242	**< 0.001**
Overweight	1.143	1.040–1.255	**0.005**	1.164	1.059–1.280	**0.002**	1.159	1.054–1.274	**0.002**
Obese	1.326	1.176–1.495	**< 0.001**	1.333	1.182–1.504	**< 0.001**	1.319	1.169–1.489	**< 0.001**
**Smoking status**									
Never–smokers	1.000			1.000			1.000		
Ex–smokers	1.029	0.865–1.226	0.744	1.019	0.854–1.215	0.836	1.013	0.849–1.208	0.886
Light smokers	1.090	0.987–1.203	0.088	1.079	0.972–1.197	0.153	1.079	0.972–1.197	0.152
Heavy smokers	1.154	0.757–1.758	0.505	1.147	0.749–1.757	0.529	1.143	0.746–1.751	0.540
**Alcohol consumption**									
Non–drinkers	1.000			1.000			1.000		
Current drinkers	0.967	0.876–1.067	0.507	0.956	0.862–1.061	0.395	0.958	0.864–1.064	0.424
**Physical activity**									
Low	1.000			1.000			1.000		
Moderate	0.834	0.748–0.929	**0.001**	0.860	0.771–0.960	**0.007**	0.866	0.776–0.968	**0.011**
High	0.964	0.877–1.060	0.451	0.968	0.880–1.065	0.504	0.981	0.891–1.081	0.704
**Type of dust**									
Coal dust	1.000			1.000			1.000		
Silica dust	1.182	1.049–1.330	**0.006**	1.167	1.034–1.316	**0.012**	1.170	1.036–1.320	**0.011**
PVC dust	1.286	1.018–1.623	**0.035**	1.328	1.050–1.679	**0.018**	1.340	1.059–1.696	**0.015**
Other dusts	0.926	0.833–1.029	0.151	0.950	0.853–1.057	0.343	0.951	0.855–1.059	0.364
**Industry**									
Mining	1.000			1.000			1.000		
Manufacturing	0.287	0.173–0.474	**< 0.001**	0.347	0.209–0.577	**< 0.001**	0.354	0.213–0.588	**< 0.001**
Other industries	0.162	0.096–0.273	**< 0.001**	0.202	0.120–0.342	**< 0.001**	0.207	0.122–0.350	**< 0.001**
**Enterprise scale**									
Large or medium	1.000			1.000			1.000		
Small or micro	1.650	1.490–1.827	**< 0.001**	1.492	1.340–1.662	**< 0.001**	1.503	1.348–1.676	**< 0.001**
**Occupation**									
Mixed laborer	1.000			1.000			1.000		
Mental laborer	1.423	1.080–1.875	**0.012**	1.508	1.142–1.991	**0.004**	1.469	1.111–1.944	**0.007**
Physical laborer	1.444	1.323–1.575	**< 0.001**	1.332	1.218–1.458	**< 0.001**	1.336	1.219–1.464	**< 0.001**
**Length of service (years)**									
≤ 5	1.000			1.000			1.000		
5–10	0.849	0.764–0.942	**0.002**	0.865	0.776–0.964	**0.009**	0.873	0.783–0.972	**0.014**
>10	0.595	0.539–0.658	**< 0.001**	0.624	0.559–0.695	**< 0.001**	0.626	0.561–0.698	**< 0.001**
**Working shift**									
Day shifts	1.000			1.000			1.000		
Night or rotating shifts	1.097	1.005–1.197	**0.039**	1.025	0.937–1.121	0.585	1.042	0.952–1.140	0.373
**Monthly income (RMB)**									
< 5,000	1.000			1.000			1.000		
5,000–9,999	1.255	1.147–1.373	**< 0.001**	1.300	1.187–1.424	**< 0.001**	1.304	1.190–1.429	**< 0.001**
≥10,000	1.885	1.397–2.544	**< 0.001**	1.915	1.414–2.592	**< 0.001**	1.880	1.385–2.550	**< 0.001**
**Respiratory protection**									
Yes	1.000			1.000			1.000		
No	1.154	1.038–1.284	**0.008**	1.167	1.048–1.299	**0.005**	1.169	1.049–1.302	**0.005**

BMI, body mass index; OR, odds ratio; 95% CI, 95% confidence interval; FVC, forced vital capacity; PVC dust, polyvinyl chloride dust.

Bold indicates that the *P*-value is < 0.05, indicating statistical significance.

**Table 6:** The risk of abnormal FEV_1_/FVC among employees with different features.

**Variables**	**Model 1**			**Model 2**			**Model 3**		
	**OR**	**95% CI**	***P***	**OR**	**95% CI**	***P***	**OR**	**95% CI**	***P***
**Gender**									
Female	1.000			1.000			1.000		
Male	0.887	0.790–0.996	**0.042**	0.891	0.776–1.022	0.099	0.870	0.758–1.000	**0.049**
**Age (years)**									
≤ 50	1.000			1.000			1.000		
>50	1.261	1.079–1.474	**0.003**	1.468	1.240–1.738	**< 0.001**	1.445	1.219–1.713	**< 0.001**
**Education level**									
Junior college or above	1.000			1.000			1.000		
High school or technical secondary school	0.641	0.571–0.720	**< 0.001**	0.640	0.569–0.720	**< 0.001**	0.646	0.574–0.728	**< 0.001**
Junior high school or below	0.548	0.479–0.627	**< 0.001**	0.546	0.476–0.627	**< 0.001**	0.555	0.482–0.638	**< 0.001**
**Marital status**									
Married	1.000			1.000			1.000		
Unmarried	1.046	0.936–1.169	0.425	0.943	0.840–1.058	0.317	0.965	0.859–1.085	0.553
Divorced or widowed	0.836	0.654–1.068	0.152	0.891	0.696–1.142	0.363	0.893	0.697–1.144	0.371
**BMI**									
Normal weight	1.000			1.000			1.000		
Underweight	0.865	0.633–1.182	0.362	0.800	0.584–1.097	0.166	0.805	0.587–1.104	0.179
Overweight	1.040	0.932–1.161	0.484	1.040	0.930–1.163	0.490	1.039	0.930–1.162	0.497
Obese	1.090	0.950–1.251	0.221	1.089	0.948–1.252	0.227	1.086	0.945–1.248	0.247
**Smoking status**									
Never-smokers	1.000			1.000			1.000		
Ex-smokers	1.034	0.847–1.264	0.741	1.075	0.878–1.316	0.486	1.081	0.883–1.324	0.451
Light smokers	0.891	0.793–1.001	0.051	0.912	0.807–1.031	0.140	0.911	0.806–1.029	0.135
Heavy smokers	1.545	0.998–2.392	0.051	1.586	1.019–2.471	**0.041**	1.569	1.007–2.445	**0.047**
**Alcohol consumption**									
Non–drinkers	1.000			1.000			1.000		
Current drinkers	1.071	0.955–1.202	0.239	1.095	0.970–1.236	0.144	1.083	0.959–1.223	0.197
**Physical activity**									
Low	1.000			1.000			1.000		
Moderate	1.136	1.004–1.287	**0.044**	1.083	0.955–1.228	0.212	1.086	0.957–1.232	0.199
High	0.915	0.819–1.023	0.120	0.913	0.816–1.022	0.113	0.917	0.819–1.027	0.136
**Type of dust**									
Coal dust	1.000			1.000			1.000		
Silica dust	0.485	0.420–0.560	**< 0.001**	0.503	0.435–0.582	**< 0.001**	0.498	0.430–0.576	**< 0.001**
PVC dust	0.235	0.160–0.346	**< 0.001**	0.226	0.153–0.333	**< 0.001**	0.229	0.155–0.337	**< 0.001**
Other dusts	0.874	0.779–0.982	**0.024**	0.878	0.780–0.988	**0.031**	0.868	0.771–0.978	**0.020**
**Industry**									
Mining	1.000			1.000			1.000		
Manufacturing	1.517	0.875–2.631	0.138	1.215	0.698–2.117	0.491	1.216	0.698–2.119	0.491
Other industries	5.815	3.305–10.231	**< 0.001**	4.395	2.484–7.774	**< 0.001**	4.372	2.470–7.740	**< 0.001**
**Enterprise scale**									
Large or medium	1.000			1.000			1.000		
Small or micro	1.136	1.014–1.272	**0.028**	1.413	1.250–1.596	**< 0.001**	1.394	1.233–1.577	**< 0.001**
**Occupation**									
Mixed laborer	1.000			1.000			1.000		
Mental laborer	0.693	0.507–0.946	**0.021**	0.646	0.472–0.884	**0.006**	0.644	0.469–0.883	**0.006**
Physical laborer	0.469	0.425–0.518	**< 0.001**	0.509	0.460–0.564	**< 0.001**	0.512	0.461–0.568	**< 0.001**
**Length of service (years)**									
≤ 5	1.000			1.000			1.000		
5–10	1.070	0.944–1.212	0.292	1.097	0.963–1.249	0.162	1.094	0.960–1.246	0.177
>10	1.551	1.384–1.738	**< 0.001**	1.543	1.361–1.750	**< 0.001**	1.531	1.349–1.737	**< 0.001**
**Working shift**									
Day shifts	1.000			1.000			1.000		
Night or rotating shifts	0.805	0.727–0.890	**< 0.001**	0.859	0.775–0.952	**0.004**	0.855	0.771–0.948	**0.003**
**Monthly income (RMB)**									
< 5,000	1.000			1.000			1.000		
5,000–9,999	0.568	0.512–0.630	**< 0.001**	0.551	0.497–0.612	**< 0.001**	0.555	0.500–0.617	**< 0.001**
≥10,000	0.347	0.232–0.521	**< 0.001**	0.342	0.227–0.514	**< 0.001**	0.332	0.220–0.500	**< 0.001**
**Respiratory protection**									
Yes	1.000			1.000			1.000		
No	1.049	0.929–1.186	0.439	0.996	0.880–1.128	0.955	0.981	0.866–1.111	0.760

BMI, body mass index; OR, odds ratio; 95% CI, 95% confidence interval; FVC, forced vital capacity; FEV_1_, forced expiratory volume in 1 s; PVC dust, polyvinyl chloride dust.

Bold indicates that the *P*-value is < 0.05, indicating statistical significance.

The risk of abnormality in FEV_1_ was higher in employees with a high school or technical secondary school education [1.156 (1.046–1.277)], employees with a junior high school education or below [1.319 (1.177–1.479)], mental laborers [1.343 (1.017–1.773)], physical laborers [1.166 (1.064–1.277)], individuals with a monthly income of 5,000–9,999 RMB [1.135 (1.036–1.243)], and those with a monthly income of ≥ 10,000 RMB [1.612 (1.206–2.155)] in Model 3. Similarly, for FVC, the corresponding ORs were 1.276 (1.154–1.411), 1.695 (1.508–1.904), 1.469 (1.111–1.944), 1.336 (1.219–1.464), 1.304 (1.190–1.429), and 1.880 (1.385–2.550) in Model 3, respectively. Conversely, for FEV_1_/FVC, the corresponding ORs were 0.646 (0.574–0.728), 0.555 (0.482–0.638), 0.644 (0.469–0.883), 0.512 (0.461–0.568), 0.555 (0.500–0.617), and 0.332 (0.220–0.500) in Model 3, respectively, demonstrating a lower risk of FEV_1_/FVC abnormalities.

Regarding physical activity, moderate physical activity could reduce the rate of abnormal FEV_1_ with an OR of 0.884 (0.793–0.985) in Model 1 and FVC with an OR of 0.866 (0.776–0.968) in Model 3 compared to low physical activity. Conversely, individuals engaging in moderate physical activity had a significantly higher rate of abnormal FEV_1_/FVC than those engaging in low physical activity, with an OR of 1.136 (1.004–1.287) in Model 1. Compared to employees who worked day shifts, those who worked night or rotating shifts exhibited a higher risk of abnormal FEV_1_ with an OR of 1.149 (1.051–1.257) in Model 3 and FVC with an OR of 1.097 (1.005–1.197) in Model 1. However, they exhibited a lower risk of abnormal FEV_1_/FVC with an OR of 0.855 (0.771–0.948) in Model 3.

Three models consistently indicated that marital status and BMI had an effect on the risk of abnormal FEV_1_ and FVC. The risk of abnormality in FEV_1_ was higher in unmarried individuals [1.370 (1.240–1.513)] compared to married individuals in Model 3. Similarly, for FVC, the corresponding OR was 1.264 (1.143–1.398) in Model 3. Individuals who were underweight [1.467 (1.131–1.904)] or obese [1.241 (1.101–1.398)] were more likely to have an increased risk of abnormal FEV_1_ compared to those with a normal weight in Model 3. Similarly, being underweight [1.719 (1.317–2.242)], overweight [1.159 (1.054–1.274)], or obese [1.319 (1.169–1.489)] may increase the risk of FVC abnormalities in Model 3. In the fully adjusted model, heavy smokers [1.569 (1.007–2.445)] had a higher risk of abnormal FEV_1_/FVC compared to never-smokers, and the absence of respiratory protective equipment [1.169 (1.049–1.302)] significantly increased the risk of abnormal FVC.

In the fully adjusted model, compared to coal dust, silica dust and PVC dust increased the risk of abnormal FVC with ORs of 1.170 (1.036–1.320) and 1.340 (1.059–1.696), respectively, but decreased the risk of FEV_1_/FVC with ORs of 0.498 (0.430–0.576) and 0.229 (0.155–0.337), respectively. In addition, individuals exposed to other dusts had a decreased risk of abnormal FEV_1_ with an OR of 0.877 (0.790–0.974) in Model 1 and a similarly decreased risk of FEV_1_/FVC abnormalities with an OR of 0.868 (0.771–0.978) in Model 3. Compared to individuals in mining in Model 3, employees in manufacturing had lower abnormal risks of FEV_1_ and FVC with ORs of 0.651 (0.431–0.983) and 0.354 (0.213–0.588), respectively. Similarly, employees in other industries had lower abnormal risks of FEV_1_ and FVC with ORs of 0.555 (0.359–0.857) and 0.207 (0.122–0.350), respectively, while they had a higher risk of abnormal FEV_1_/FVC with an OR of 4.372 (2.470–7.740).

Regarding the enterprise scale, compared to individuals in large or medium enterprises in Model 3, employees in small or micro enterprises had higher abnormal risks of FEV_1_, FVC, and FEV_1_/FVC with ORs of 1.227 (1.104–1.364), 1.503 (1.348–1.676), and 1.394 (1.233–1.577), respectively. Surprisingly, compared to individuals with ≤ 5 years of service in Model 3, those with 5–10 years of service had lower risks of abnormal FEV_1_ and FVC with ORs of 0.846 (0.760–0.941) and 0.873 (0.783–0.972), respectively. Similarly, individuals with >10 years of service had lower abnormal risks of FEV_1_ and FVC with ORs of 0.660 (0.593–0.736) and 0.626 (0.561–0.698), respectively, while they had a higher risk of abnormal FEV_1_/FVC with an OR of 1.531 (1.349–1.737).

## 4 Discussion

In this cross-sectional study, we analyzed the status and possible influencing factors of lung ventilation function among employees exposed to dust in enterprises located in the Eighth Division, XPCC, China. According to the fixed value criteria, the abnormal rates of FEV_1_, FVC, and FEV_1_/FVC were 31.6, 1.4, and 0.4%, respectively, which were lower than the rates of abnormal lung ventilation function indicators among textile mill workers in Myanmar, with abnormal rates of 34.3, 36.7, and 3.9% for FEV_1_, FVC, and FEV_1_/FVC, respectively (18). It can be seen that the XPCC attaches great importance to the health protection of the occupational population. Additionally, despite the fact that the adoption of the LLN criteria might overestimate the abnormal rates of lung ventilation function, the LLN criteria are more scientific and recommended for use in epidemiological studies (36) due to partly avoiding a high false negative rate in younger populations and over diagnosis in older populations associated with the fixed value criteria (37). Therefore, the LLN criteria were utilized in this study to assess the status of lung ventilation function and explore its influencing factors.

According to the findings of this study, gender, age, education level, marital status, BMI, smoking status, physical activity, the type of dust, industry, enterprise scale, occupation, length of service, working shift, monthly income, and respiratory protection were identified as influencing factors for lung ventilation function. Males, individuals with lower educational levels, unmarried, underweight, overweight, and obese individuals were more likely to experience lung ventilatory function abnormalities. In addition, heavy smokers had a higher risk of abnormal FEV_1_/FVC than never-smokers. Moderate levels of physical activity were favorable for improving individuals' lung ventilation function. Silica dust and PVC dust were more likely to trigger lung ventilation dysfunction than coal dust. Employees working in mining and those employed in small and micro enterprises were at higher risk of lung ventilation dysfunction. Engaging in night or shift work could increase the risk of abnormal lung ventilation dysfunction. Mental laborers and physical laborers had a higher risk of developing abnormal lung ventilation function than mixed laborers. Employees aged >50, individuals with a monthly income of < 5,000 RMB, individuals with 5–10 years of service, and those with >10 years of service had a lower risk of abnormal lung ventilation function. In addition, wearing respiratory protection equipment was effective in reducing the risk of abnormal lung ventilation function. Notably, the direction of influence from factors such as gender on FEV_1_/FVC was opposite to their influence on FEV_1_ and FVC, which could be attributed to the possibility that FEV_1_ and FVC were affected to varying degrees by these factors.

Exposure to dust can worsen lung function, which is a major contributing factor in this process (25). The type of dust is one of the most characteristic occupational features of employees, and different types of dust vary in their damaging effects on lung function. One study noted that silica dust caused more severe damage to the lungs compared to composite epoxy dust (38). In this study, both silica dust and PVC dust were found to increase the risk of abnormal FVC compared to coal dust. However, the opposite result occurred for FEV_1_/FVC.

It is well-known that silica dust is an inorganic dust that is smaller, harder, and more irregular than organic dust, making it easier to enter the respiratory system and cause mechanical injury to the lungs. In addition, silica dust, which is rich in silicon dioxide, can damage lung macrophages, ultimately causing the development of lung fibrosis (39). It is worth noting that the higher number of surface area atoms per unit mass of PVC dust greatly increases the surface area available for chemical reactions with body fluids and tissues that come into contact with it (40). Hence, PVC dust may lower FVC, artificially raise the FEV_1_/FVC ratio, and even lead to lung fibrosis or cancer (41). Compared to coal dust, other dusts, mainly some unidentified types of dust, reduced the abnormal rates of FEV_1_ and FEV_1_/FVC, which might be influenced by factors such as the concentration of the dust.

In general, different industries involve varying types and concentrations of dust, resulting in different levels of lung ventilation function among employees. We found that, compared to employees in mining, those in manufacturing and other industries were at a lower risk of impaired lung ventilation function, which could be attributed to higher concentrations of coal dust in mining. Therefore, close attention should be paid to lung ventilation function among individuals exposed to dust in mining.

Notably, we observed that wearing respiratory protective equipment during work was beneficial in impeding the decline in lung ventilation function, which was supported by other studies. For instance, firefighters who frequently wore respiratory protective devices were more likely to experience a normal rate of decline in lung function compared to their counterparts without respiratory protection (42). Due to this, it is necessary for employers to regularly remind individuals exposed to dust to wear protective masks appropriately.

In this study, individuals exposed to dust with lower educational levels had poorer lung ventilation function. This finding was supported by another study, which revealed that flour mill workers with primary education were more prone to experiencing chronic respiratory symptoms compared to those with secondary school education and above (43). One possible reason is that individuals with lower educational levels may have been assigned to jobs with dust exposure and may lack knowledge regarding the health hazards of dust and how to protect themselves from its effects. Therefore, it is crucial to popularize knowledge and skills for protecting lung ventilation function among employees exposed to dust.

Standard morphometric measures have confirmed that, compared to females with the same weight and height, males have larger lungs, a higher number of bronchi, greater alveolar surface area, and wider airway caliber (44). This suggests that males theoretically have better lung ventilation function than females. Some scholars pointed out that male individuals had higher FEV_1_ and FVC than females (45). Despite this, there are differences in the rate of decline in lung ventilation function between male and female individuals.

One study noted gender-related differences in lung ventilation function, with higher annual decline rates in males than in females (46), which indicated that males were more prone to experiencing a decrease in their lung ventilation function below normal levels, exhibiting an anomalous state. Similarly, in the present study, the risk of abnormal FEV_1_ and FVC was significantly higher in males than in females. Three possible explanations could explain this observation. Firstly, the more significant decline in respiratory muscle strength among males compared to females might be an important factor contributing to this phenomenon (44). Secondly, the decline in lung ventilation function might be positively proportional to lung size, which is related to the differences in airway caliber between males and females (47). Finally, taking a shower and changing work clothes after work were more common among females, which reduced further damage to their lung ventilation function.

Smoking is also known to induce oxidative stress and inflammatory states, leading to airway hyper responsiveness, subsequent airway stenosis, and increased airway resistance, ultimately resulting in weakened lung function (48, 49). A study revealed that active smokers exhibited significantly lower values for FEV_1_, FVC, and FEV_1_/FVC compared to never-smokers (50). Some scholars also indicated that ex-smokers and current smokers experienced an accelerated decline in lung function compared to never-smokers (51). In this study, compared to never-smokers, heavy smokers had a higher prevalence of abnormal FEV_1_/FVC. Notably, early smoking cessation could slow down the declining trend of lung function in current smokers (52). According to the Healthy China 2030 strategy, the government has set a goal of reducing the adult smoking rate from 27.7 to 20% by 2030 (53), which will safeguard the lung function of individuals.

Some scholars revealed that appropriate physical activity could enhance immune function by increasing the number of natural killer cells and lymphocytes (54). Similarly, in this study, individuals engaging in moderate physical activity exhibited a significantly lower rate of abnormal lung ventilation function than those engaging in low physical activity. Therefore, it is advisable to emphasize the importance of ensuring daily physical exercise during non-working hours for employees exposed to dust.

Individuals can benefit from being in a healthy marital status. Specifically, for married individuals, their spouses can often remind them to maintain healthy behaviors and provide them with encouragement (55), and they also have dual incomes within their households, which contributes to their less life pressures and better mental health. Notably, there is a connection between mental health and lung ventilation function. Some scholars found that depression could lower FEV_1_ (56) and FVC (57) in individuals by increasing levels of pro-inflammatory cytokines (58). The above mechanism could explain the observed decrease in FEV_1_ and FVC among unmarried individuals compared to married individuals in the present study.

In many studies, an association between BMI and lung ventilation function can be generally observed. A study revealed that, compared to individuals with a normal weight, overweight and obese individuals were associated with decreased absolute values of FEV_1_ and FVC (59). The present study demonstrated an increased risk of abnormal lung ventilation function among underweight, overweight, and obese individuals. As we all know, weight gain results in the diaphragm being pushed upward and fat accumulation, which will cause incomplete lung expansion during inhalation and restricted airflow (60, 61). In addition, adipose tissue can secrete various inflammatory factors (62), thereby narrowing airway diameter and impairing lung function (63). Notably, being underweight implies compromised nutritional status, which is associated with lung dysfunction (64). Therefore, it is advisable to maintain a normal weight, which is a controllable factor in preventing lung ventilation function impairment.

Night or rotating shifts have been found to impair both physical and mental health, including lung ventilation function. For instance, research has shown an increased risk of abnormal FEV_1_ among individuals engaged in night or rotating shifts (65). The same phenomenon was observed in the present analysis. The possible reason is that individuals engaged in night or rotating shifts have fewer daytime physical activities and more common nocturnal eating behaviors (66).

Regarding the enterprise scale, research indicated that employees in small and micro enterprises had a higher prevalence of abnormal FEV_1_, FVC, and FEV_1_/FVC than those in medium enterprises (67). In this analysis, we observed a significant increase in the risk of FEV_1_, FVC, and FEV_1_/FVC abnormalities among employees in small or micro enterprises compared to large or medium enterprises, which indicated that large and medium enterprises paid more attention to occupational health.

In this analysis, mental and physical laborers were found to have worse lung ventilation function compared to mixed laborers. Evidently, physical laborers had higher levels of physical activity, higher respiratory rates, and were more prone to inhaling dust during work compared to mixed laborers, consequently impairing their lung ventilation function. Notably, sedentary behavior is more prevalent in mental laborers, primarily due to their office-based work, compared to mixed laborers, which can cause a decline in lung ventilation function by increasing the risk of obesity (68).

Regarding the length of service and age, some scholars found that both length of service (69) and age (44) were negatively related to lung ventilation function. However, the results of this study were markedly different, which could be explained by the healthy worker effect. It is possible that only individuals with better lung ventilation function will continue working in an environment with dust due to the presence of health examinations and safety standards. In addition, this phenomenon may be because the employers from these enterprises prefer to arrange for healthier young individuals to work night or rotating shifts, which reflects an aspect of humanized management by these enterprises.

In this present study, employees with a higher monthly income exhibited a higher prevalence of lung ventilation dysfunction. One possible reason is that individuals with financial pressures tend to choose occupations with a higher monthly income, which may cause them to work in environments with higher concentrations of dust, thereby causing their worse lung ventilation function.

Moderate alcohol intake could increase FEV_1_ and FVC improve lung function (70) potentially by enhancing mucociliary clearance, stimulating bronchodilation, and even alleviating airway damage (59). In contrast, chronic alcohol exposure often leads to hepatopulmonary syndrome, which can cause respiratory impairment (71). In this study, univariate analysis revealed a statistically significant association between lung ventilation function and alcohol consumption, while multivariable analysis showed the opposite finding. One possible explanation is that there is a “spurious association” in the univariate analysis that can easily vanish by adjusting for the relevant confounding factors.

Occupational harmful factors can affect an individual's lung health and lead to the development of occupational diseases. Notably, some scholars have begun to pay attention to the prognosis of occupational diseases and their impact on the employment status of individuals. Mason et al. found that inhalation of diisocyanates could induce occupational asthma, which was found to be attenuated in some patients during follow-up, and being diagnosed at a younger age was a major predictor of a good prognosis (72). Similarly, one study found that workers with alleviated asthma were younger at the time of diagnosis, had a shorter duration of symptom exposure, better lung function, and lower bronchial hyper-responsiveness than individuals with persistent occupational asthma, and that occupational asthma led to a change in job position in 43.8% of individuals (73). Hence, for the working population, early detection of health impairments, prompt treatment, and necessary adjustments in jobs are essential for effectively managing the condition, improving prognosis, and enhancing their overall health status.

## 5 Limitations

There are still some limitations to this study. Firstly, this study was a cross-sectional study, which cannot determine a causal relationship between lung ventilation function and relevant influencing factors. Secondly, self-administered questionnaire could lead to possible recall bias and decreased objectivity. Thirdly, the lung ventilation function measurement was only partial, lacking data on global spirometry and results from chest imaging techniques. This prevents further considerations on the type of respiratory abnormality found (e.g., obstructive vs. restrictive abnormality). In addition, healthy worker effect may underestimate the influence of certain factors on lung ventilation function. Finally, this study lacked data such as the concentration of dust, so we are unclear about their association with lung ventilation function. Further prospective studies are needed to overcome these limitations and identify more controllable factors affecting lung ventilation function of employees exposed to dust.

## 6 Conclusion

This study revealed a relatively low prevalence of abnormal lung ventilation function among employees exposed to dust in enterprises located in the Eighth Division, XPCC, China. Several potential factors associated with lung ventilation function abnormalities were identified, including gender, age, education level, marital status, BMI, smoking status, physical activity, the type of dust, industry, enterprise scale, occupation, length of service, working shift, monthly income, and respiratory protection. It can be seen that enhancing personal protection, reducing smoking, and maintaining a normal weight are beneficial for protecting the lung ventilation function of employees exposed to dust in this region.

## Data availability statement

The original contributions presented in the study are included in the article/supplementary material, further inquiries can be directed to the corresponding authors.

## Ethics statement

The studies involving humans were approved by the Science and Technology Ethical Committee of the First Affiliated Hospital, School of Medicine, Shihezi University (Ethical Review Number: KJX2022-104-01). The studies were conducted in accordance with the local legislation and institutional requirements. The participants provided their written informed consent to participate in this study.

## Author contributions

YZ: Data curation, Formal analysis, Methodology, Writing—original draft, Writing—review & editing. SZ: Data curation, Formal analysis, Methodology, Writing—original draft, Writing—review & editing. JL: Data curation, Formal analysis, Methodology, Writing—original draft, Writing—review & editing. RD: Writing—original draft. QW: Writing—original draft. GS: Funding acquisition, Writing—review & editing. YH: Writing—review & editing.
